# Exploratory assessment of the effect of systemic administration of soluble glycoprotein 130 on cognitive performance and chemokine levels in a mouse model of experimental traumatic brain injury

**DOI:** 10.1186/s12974-024-03129-0

**Published:** 2024-06-05

**Authors:** Ian G. Gober, Ashley L. Russell, Tyler J. Shick, Vincent A. Vagni, Jenna C. Carlson, Patrick M. Kochanek, Amy K. Wagner

**Affiliations:** 1 Department of Physical Medicine and Rehabilitation, School of Medicine, University of Pittsburgh, 3471 Fifth Avenue, Suite 910, Pittsburgh, PA 15213 USA; 2 Safar Center for Resuscitation Research, John G. Rangos Research Center, Pittsburgh, PA USA; 3 Department of Critical Care Medicine, School of Medicine, University of Pittsburgh, Pittsburgh, PA USA; 4 Center for Neuroscience, School of Medicine, University of Pittsburgh, Pittsburgh, PA USA; 5 Department of Biostatistics, School of Public Health, University of Pittsburgh, Pittsburgh, PA USA; 6 Department of Human Genetics, School of Public Health, University of Pittsburgh, Pittsburgh, PA USA; 7 Department of Neuroscience, School of Arts and Sciences, University of Pittsburgh, Pittsburgh, PA USA; 8 Clinical and Translational Science Institute, University of Pittsburgh, Pittsburgh, PA USA

**Keywords:** Traumatic brain injury, Interleukin-6, Trans-signaling, Soluble glycoprotein-130, Morris water maze, Cognition, Anxiety, Chemokines

## Abstract

Uncontrolled neuroinflammation mediates traumatic brain injury (TBI) pathology and impairs recovery. Interleukin-6 (IL-6), a pleiotropic inflammatory regulator, is associated with poor clinical TBI outcomes. IL-6 operates via classical-signaling through membrane-bound IL-6 receptor (IL-6R) and trans-signaling through soluble IL-6 receptor (s)IL-6R. IL-6 trans-signaling specifically contributes to neuropathology, making it a potential precision therapeutic TBI target. Soluble glycoprotein 130 (sgp130) prevents IL-6 trans-signaling, sparing classical signaling, thus is a possible treatment. Mice received either controlled cortical impact (CCI) (6.0 ± 0.2 m/s; 2 mm; 50-60ms) or sham procedures. Vehicle (VEH) or sgp130-Fc was subcutaneously administered to sham (VEH or 1 µg) and CCI (VEH, 0.25 µg or 1 µg) mice on days 1, 4, 7, 10 and 13 post-surgery to assess effects on cognition [Morris Water Maze (MWM)] and ipsilateral hemisphere IL-6 related biomarkers (day 21 post-surgery). CCI + sgp130-Fc groups (0.25 µg and 1 µg) were combined for analysis given similar behavior/biomarker outcomes. CCI + VEH mice had longer latencies and path lengths to the platform and increased peripheral zone time versus Sham + VEH and Sham + sgp130-Fc mice, suggesting injury-induced impairments in learning and anxiety. CCI + sgp130-Fc mice had shorter platform latencies and path lengths and had decreased peripheral zone time, indicating a therapeutic benefit of sgp130-Fc after injury on learning and anxiety. Interestingly, Sham + sgp130-Fc mice had shorter platform latencies, path lengths and peripheral zone times than Sham + VEH mice, suggesting a beneficial effect of sgp130-Fc, independent of injury. CCI + VEH mice had increased brain IL-6 and decreased sgp130 levels versus Sham + VEH and Sham + sgp130-Fc mice. There was no treatment effect on IL-6, sIL6-R or sgp130 in Sham + VEH versus Sham + sgp130-Fc mice. There was also no treatment effect on IL-6 in CCI + VEH versus CCI + sgp130-Fc mice. However, CCI + sgp130-Fc mice had increased sIL-6R and sgp130 versus CCI + VEH mice, demonstrating sgp130-Fc treatment effects on brain biomarkers. Inflammatory chemokines (MIP-1β, IP-10, MIG) were increased in CCI + VEH mice versus Sham + VEH and Sham + sgp130-Fc mice. However, CCI + sgp130-Fc mice had decreased chemokine levels versus CCI + VEH mice. IL-6 positively correlated, while sgp130 negatively correlated, with chemokine levels. Overall, we found that systemic sgp130-Fc treatment after CCI improved learning, decreased anxiety and reduced CCI-induced brain chemokines. Future studies will explore sex-specific dosing and treatment mechanisms for sgp130-Fc therapy.

**Supplementary Information:**

The online version contains supplementary material available at 10.1186/s12974-024-03129-0.

## Introduction

There are no disease-modifying therapies for traumatic brain injury (TBI) to improve neurologic recovery. TBI survivors often have lasting cognitive, behavioral, and sensory deficits, which may be linked to chronic uncontrolled inflammation. Following TBI, the pleiotropic cytokine, interleukin-6 (IL-6), is upregulated acutely and chronically (3-months) [1]. Patients with higher cerebrospinal fluid (CSF) IL-6 levels had worse 6-month global outcomes following severe TBI. Other reports demonstrate that patients with severe TBI and with higher ratios at 3 months post-injury of serum IL-6 to the anti-inflammatory cytokine IL-10 also had increased odds of worse 6-month global outcomes [2]. IL-6 contributes to other inflammatory pathologies like rheumatoid arthritis (RA), multiple sclerosis (MS), asthma, cancer, metabolic syndrome, type 2 diabetes, and inflammatory bowel disease (IBD) [3–5]. Associations with chronic disease states and poor neurologic outcomes after TBI highlight IL-6 signaling as a possible therapeutic target.

IL-6 also regulates homeostatic and anti-inflammatory processes [6] and mechanistically acts via both classical and trans-signaling. Classical signaling occurs when IL-6 binds to membrane-bound IL-6 receptor (mIL-6R) in a complex with membrane-bound glycoprotein-130 (gp130). Classical signaling only occurs in cell types containing mIL-6R, which include microglia, neutrophils, naïve T-cells, and hepatocytes [7, 8]. Also, mIL-6R activation is critically involved in microglial priming across the lifespan by enhancing both pro-inflammatory genes and major histocompatibility complex (MHC)-II expression [9]. Activating the classical signaling pathway primarily regulates metabolic and regenerative processes [6]. Conversely, trans-signaling involves soluble IL-6R (sIL-6R), which binds IL-6 before binding to the ubiquitously expressed gp130. Soluble (s)gp130 is the natural inhibitor of the agonistic IL-6/sIL-6R complex acting as a decoy receptor [10]. IL-6 has three binding sites for receptors, with site I recognizing IL-6R and sites II and III recognizing gp130. IL-6 and IL-6R antibodies target site I of the IL-6/IL-6R complex which affects all (classical, trans) IL-6 signaling. The fusion protein sgp130(-Fc) is unique in that it selectively targets sites II and III, only affecting trans-signaling and sparing classical signaling [11]. Despite the differences in upstream signaling mechanisms between classical and trans-signaling, which rely on cell-type specific differences in IL-6 receptor subtype/subunit expression, the intracellular Janus kinsase/signal transducers and activators of transcription (JAK/STAT3) signaling does not differ as a function of IL-6 mediated classical versus trans-signaling [12]. However, the different effects observed with trans-signaling are a function of the wide ranging cell types in which signaling occurs as well as stronger signaling observed compared to classical pathways [13]. For example, while classical IL-6 signaling is less able to strongly activate STAT3 in endothelial cells, classical signaling patterns promote cell survival, while trans-signaling patterns in endothelial cells can promote inflammatory activation [14]. Relevant to TBI, research also suggests that IL-6 trans-signaling involving endothelial cells facilitates intercellular adhesion molecule (ICAM) and monocyte chemoattractant protein (MCP)-1 secretion, while classical IL-6 signaling facilitates IL-8 mediated immune cell transmigration [14]. Broadly speaking, IL-6 trans-signaling propagates inflammation in the central nervous system (CNS), facilitating microgliosis and astrocytosis through chemokine signaling, contributing to sickness behaviors and cognitive deficits in mice, and increasing risk for neurodegenerative disorders like Alzheimer’s disease [7, 15–19].

IL-6 has potential as a therapeutic target for inflammatory diseases. For example, IL-6 deficient mice have decreased susceptibility to experimental autoimmune encephalomyelitis [4]. Also, anti-IL-6 antibody treatment (Tocilizumab) decreases symptomology in conditions such as RA, and COVID-19 [20, 21]. While proven to be beneficial, IL-6 pan-inhibition by anti-IL-6 antibody treatment affects both trans- and classical signaling causing immunosuppression. sgp130 selectively neutralizes sIL-6R, and thus, prevents trans-signaling, while maintaining the beneficial functions of classical signaling. Importantly, recent studies suggest that treatment with a sgp130 fusion protein (sgp130-Fc) can promote remission for arthritis and IBD [3, 22, 23].

Despite these findings in lipopolysaccharide (LPS)-induced inflammation models, in vitro endothelial cell models, as well as in infectious and systemic autoimmune diseases, there is limited literature regarding the translational potential of inhibiting IL-6 trans-signaling in acquired brain injury, including TBI. Therefore, we aimed to determine if selectively inhibiting IL-6 trans-signaling via systemic sgp130-Fc administration would promote behavioral and neuroinflammatory benefits in a mouse model of severe TBI.

## Materials and methods

### Animals

**Fig. 1:**
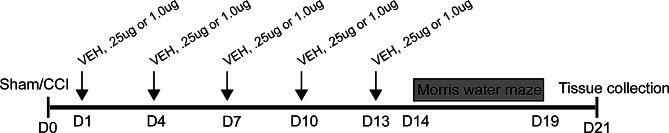
Experimental design. Mice were randomly assigned to receive either sham or CCI [day (D)0]. Starting on D1, mice received either vehicle (PBS; Sham and CCI), 0.25 µg sgp130-Fc (CCI) or 1.0 µg sgp130-Fc (Sham and CCI). Mice were treated every 3 days with vehicle or sgp130-Fc. On D14-19 mice underwent Morris water maze (MWM) testing and on D21 tissue was collected for analysis

Adult (12–15 weeks) male C57BL/6J mice (*N* = 75; Jackson Laboratory) were housed in a temperature-, humidity-, and lighting-controlled environment with *ad libitum* access to food and water. Mice were randomly assigned to one of five groups: (1) Sham + Vehicle (Sham + VEH; *n* = 21), (2) Sham + 1 µg sgp130-Fc (Sham + 1 µg; *n* = 8), (3) controlled cortical impact (CCI) + VEH (*n* = 26), (4) CCI + 0.25 µg sgp130-Fc (CCI + 0.25 µg; *n* = 10), (5) CCI + 1 µg sgp130-Fc (CCI + 1 µg; *n* = 10). Mice in the CCI + 0.25 µg and CCI + 1 µg sgp130-Fc groups had similar treatment-associated behavioral and brain IL-6 related chemokine outcomes (Supplemental Figs. 2, 3, and 4), and therefore, were combined for all analyses (CCI + sgp130-Fc) presented in the main text. Supplemental Figs. 2, 3 and 4 present comparative findings for the CCI + 0.25 µg and CCI + 1 µg outcomes. A study design chart is provided in Fig. 1.

Mice received subcutaneous administration of either recombinant mouse sgp130-Fc chimera protein (R&D Systems) dissolved in 0.1 M phosphate-buffered saline (PBS) or VEH (PBS) on days (D) 1, 4, 7, 10, and 13 post-surgery. The sgp130-Fc dosing strategy (every 3 days) was chosen due to the 72-hour half-life of the sgp130-Fc molecule [24]. The 2 doses (0.25 µg and 1 µg) administered in this exploratory study were based on previous work. Given that the use of systemic sgp130-Fc for TBI is novel, doses were chosen to fall within the effective dosing range in other published mouse studies identified at study inception [25–27].

### Controlled cortical impact (CCI)

CCI mice were anesthetized with 4% isoflurane (induction) and 1–2% (maintenance) in 2:1 nitrous oxide/oxygen [28, 29]. A 5 mm craniotomy was made over the left parietotemporal cortex. Temperature was maintained at 37 ± 0.5ºC (rectal) during surgery. CCI was performed using a 3 mm flat-tip pneumatic impactor (velocity 6.0 ± 0.2 m/s; vertical depth 2 mm; duration 50-60ms). After impact, the bone flap was replaced, sealed with dental cement, and the incision sutured. Mice were removed from anesthesia and recovered for 30 minutes (min) with supplemental oxygen. Shams underwent all procedures except for the CCI. Three mice died, none of which received sgp130-Fc treatment. This injury severity is accompanied by impairments in motor and cognitive tasks such as Morris water maze (MWM) learning and memory as well as a lasting brain inflammatory response [30–32].

### Morris water maze (MWM)

MWM testing [28] consisted of two phases: (1) learning acquisition (D14-18) and (2) visible platform (VP; D19). Testing was conducted in a circular pool (67 cm diameter, 58 cm deep) filled with water (20–22 °C, 28 cm deep) in a room surrounded by extra-maze visual cues on the walls. During acquisition, mice underwent four trials that were randomly assigned to one of four starting locations (north, south, east, west) in the pool. A 9 cm circular platform was submerged 0.5 cm below the water surface in the southwest quadrant. Mice were given a maximum of 120 s (sec) to locate the submerged platform per trial. Mice were given 4 min inter-trial intervals in a 37 °C incubator. For VP testing, the platform was elevated 0.5 cm from the water surface. Mice were randomly assigned to one of the four starting coordinates for four trials and given a maximum of 120 s per trial. Mice were given 4 min inter-trial intervals in a 37 °C incubator. Latency and swim tracking were recorded using AnyMaze software (Stoelting Co).

### Brain luminex assay

On day 21, mice were anesthetized with 4% isoflurane and sacrificed by decapitation [33, 34]. Brains were flash-frozen, and stored at -80 °C. The brain hemisphere ipsilateral to the contusion was processed and analyzed using mouse T-cell (Millipore, MCYTOMAG-70 K-27) and soluble cytokine receptor (Millipore, MSCRMAG-42 K-12) panels following manufacturer’s protocols. Tissue was homogenized in T-PER (Thermo Fisher Scientific; 300 mg tissue:1 mL T-PER), and supernatant was removed and diluted in PBS to achieve a final protein concentration of 6 mg/mL. Samples were assayed undiluted for the mouse T-cell panel. Samples were run at a 5 times (x) dilution for the soluble cytokine receptor panel. Concentrations were measured using a Bio-Rad LX100 and LX200 microplate readers with Bio-Rad Bio-Plex manager 6.2 software.

Luminex data were processed and subjected to a linear transformation. We adopted a trimmed mean (TM) analysis of the fluorescence intensity (FI) to address low and out of range low values through a linearization process to improve overall standard curve fit and increase the accuracy of values across the range of the standard curve [35, 36]. The TM process removed the highest and lowest 10% of the measured beads for each analyte for each sample in each well, reducing random error, and providing a more precise measurement of a given well’s FI. The TM, FI and concentration for each standard were subject to log transformation to linearize the data. A third order polynomial equation, which best fit the distribution of the log transformed data, was generated from the standards. New sample concentrations were calculated with the polynomial equation. Plates were run with intra- and inter-plate controls and indexed to one another using the inter-plate control coefficients. The mean intra-plate and mean inter-plate coefficient of variance were < 15%.

### Statistical analyses

**Fig. 2:**
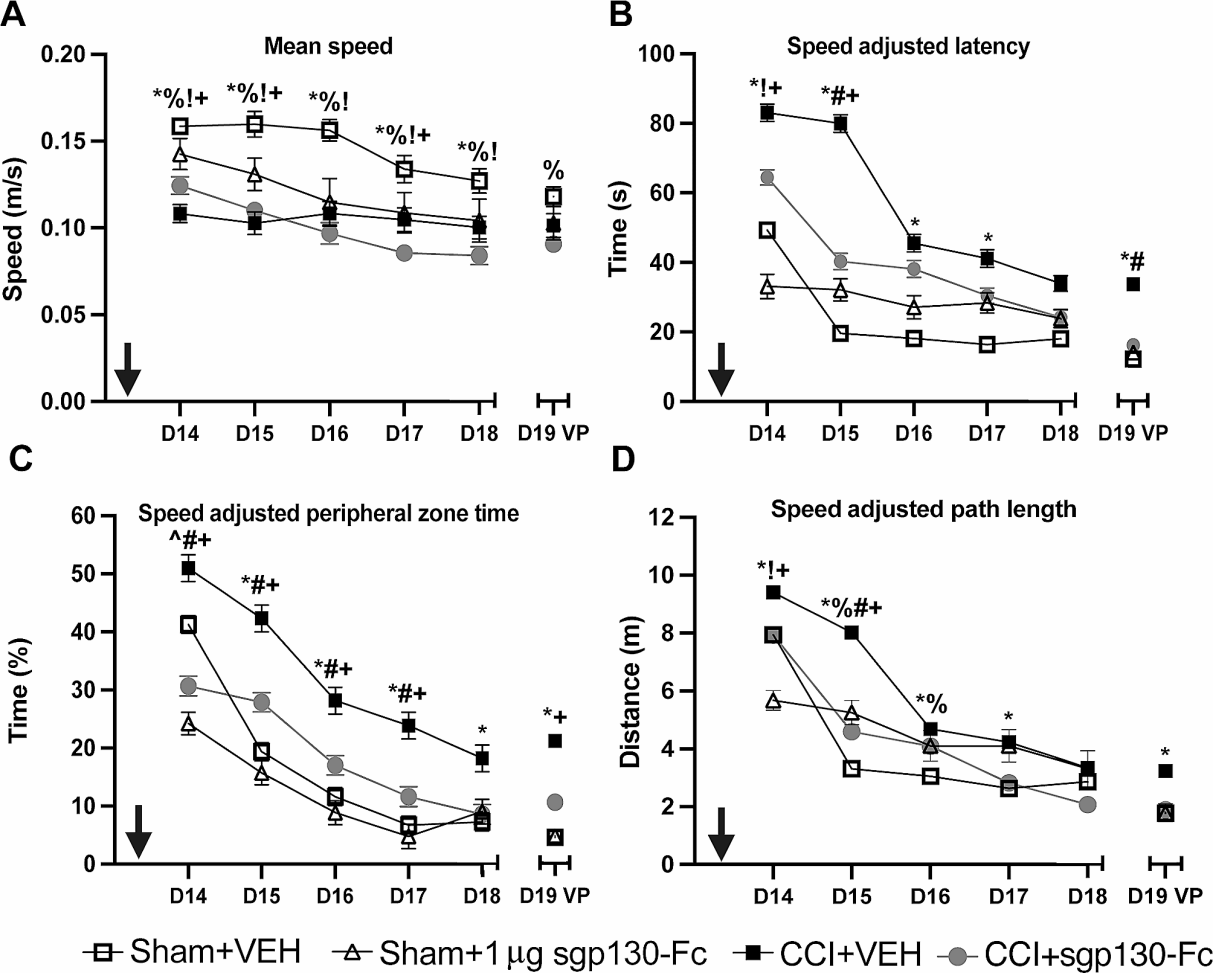
sgp130-Fc treatment post-CCI on MWM metrics during learning acquisition (D14-18) and visible platform (VP; D19). (**a**) Mean speed (**b**) speed adjusted escape latencies (**c**) speed adjusted peripheral zone time and (**d**) speed adjusted path length. Acquisition data were analyzed via mixed modeling for main effect and Sidak post-hoc testing. VP was analyzed using linear regression. Black arrow on D13 represents final sgp130-Fc or VEH administration. Lines/bars represent mean ± SEM. Significant comparisons (*p* < 0.05) include: *Sham + VEH vs. CCI + VEH, ^%^Sham + VEH vs. CCI + sgp130-Fc, ^Sham + VEH vs. Sham + 1 µg sgp130-Fc, ^#^CCI + VEH vs. CCI + sgp130-Fc, ^+^Sham + 1 µg sgp130-Fc vs. CCI + VEH, ^!^Sham + 1 µg sgp130-Fc vs. CCI + sgp130-Fc

Experimenters were blinded to treatment groups. Data were analyzed using STATA-16 or GraphPad Prism-9. Values are reported as mean ± standard error of the mean (SEM). Data were tested for distribution and variance. We used a mixed effects regression model to address the repeated measurements within the MWM assay. Mixed regression modeling is a flexible analytic approach that handles correlated data by accounting for the correlation patterns between repeated measurements within each individual to be explicitly modeled. The flexibility to accommodate both fixed effects and random effects, factors whose effects that are assumed to be the same or varied across individuals respectively, effectively captures temporal dynamics of the system to address clinically relevant questions. Within the MWM assay, a random intercept mixed effects regression model was used to generate separate intercepts for each mouse to model within-mouse variation in test performance over time. This approach incorporates mouse-to-mouse variability in the inference-making process to describe the relationship between the fixed effects and test performance. We fit models that evaluated the main effects of group, time, and the group-by-time interaction effect. We then estimated marginal means on each of MWM acquisition (D14-18) to assess the day-specific effect of group. Marginal mean contrasts with Sidak *p*-value correction were used to perform the post hoc comparisons by group. Speed adjusted mixed models were also generated (Fig. 2 and Supplemental Fig. 2) and only differed from the unadjusted models (Supplemental Fig. 1 and Supplemental Fig. 3, respectively) in that mean speed was included as a covariate to predict speed adjusted values for latency, path length and peripheral zone time. Linear regression was used to compare group relationships on VP.

Inflammatory markers were compared across groups using Kruskal-Wallis tests. Multiple comparisons adjustments were applied using Dunn’s Test. All biomarker correlations were performed with Spearman’s (r).

## Results

### Morris water maze

#### Speed-adjusted MWM outcomes

There was a main effect of group for mean swim speed (*p* < 0.001). In general, CCI mice had slower swim speeds than Sham + VEH mice across all MWM testing days (*p* < 0.05; Fig. 2A). To minimize the impact of the varying swim speeds on MWM outcomes, all MWM variables (latency, path length and peripheral zone time) were adjusted for speed to observe group-specific effects (detailed description in Methods). Group stratified correlations between swim speed and other MWM variables (D14-18) showed group differences in how swim speed affected latency, path length and peripheral zone time. Overall, latency, peripheral zone time and path length were correlated with swim speed (Table 1). These group differences provided the rationale to control for swim speed as shown in Fig. 1 and Supplemental Fig. 2 when evaluating other MWM metrics such as latency and peripheral zone time.

Table 1.

**Table 1:** MWM variables correlation with mean speed. Spearman’s (r) correlations for Morris Water Maze variables correlated with mean speed. Significant comparisons are bolded and indicate *p* < 0.05. Italics indicates a trend towards significance (*p* < 0.1)

TEST GROUP AND VARIABLE	Spearman *r*	*P*-VALUE
**SHAM + VEHICLE**		
** LATENCY**	0.1628	*0.097*
** PATH LENGTH**	0.3385	**0.0004**
** Peripheral Zone Time**	0.1937	**0.0477**
**SHAM + SGP130-FC**		
** LATENCY**	0.5163	**0.0006**
** PATH LENGTH**	0.6545	**< 0.0001**
** Peripheral Zone Time**	0.6962	**< 0.0001**
**CCI + VEHICLE**		
** LATENCY**	-0.2008	**0.022**
** PATH LENGTH**	0.2032	**0.0204**
** Peripheral Zone Time**	-0.0005	0.9959
**CCI + SGP130-FC**		
** LATENCY**	*0.0973*	0.3357
** PATH LENGTH**	0.4477	**< 0.0001**
** Peripheral Zone Time**	0.3507	**0.0003**

We determined the effect of sgp130-Fc treatment after CCI on MWM learning acquisition (latency, path length) and anxiety-like (peripheral zone time [37]) behaviors (D14-19), while adjusting for swimming speed. There was a significant group effect for latency (*p* < 0.0001), path length (*p* < 0.05), and peripheral zone time (*p* < 0.0001). CCI + VEH mice had increased latencies to platform versus Sham + VEH mice (D14-17; *p* < 0.05). CCI + sgp130-Fc mice had shorter latencies versus CCI + VEH mice [D14 (*p* = 0.076); D15 (*p* < 0.05); Fig. 2B]. The time spent in the peripheral zone was greater for CCI + VEH mice versus Sham + VEH mice (D15-18; *p* < 0.05) and CCI + sgp130-Fc mice (D14-17; *p* < 0.05; Fig. 2C). Sham + sgp130-Fc mice spent less time in the peripheral zone versus Sham + VEH mice (D14; Fig. 2C). CCI + VEH mice had increased path lengths to the hidden platform versus Sham + VEH (D14-17; *p* < 0.05) and CCI + sgp130-Fc mice (D14-15; *p* < 0.05; Fig. 2D). Speed adjusted VP performance is presented in Fig. 2B-D. Sham + VEH and CCI + sgp130-Fc mice had a shorter latency to the VP than CCI + VEH mice (*p* < 0.05; Fig. 2B). Sham + VEH mice spent less time in the peripheral zone (*p* < 0.05; Fig. 2C) and had shorter path lengths to the VP than CCI + VEH mice (*p* < 0.05; Fig. 2D).

#### Non-adjusted MWM outcomes

Analysis was also performed on MWM data without adjusting for variation in swim speed (Supplemental Fig. 1). There was a significant main effect of group for acquisition latency (*p* < 0.0001), peripheral time (*p* < 0.0001), mean speed (*p* < 0.0001) and path length (*p* < 0.05). Multiple comparison findings were similar to the speed adjusted data presented. Sham + VEH mice (D14-17 and VP; *p* < 0.05) and CCI + sgp130-Fc mice (D14-15 and VP; *p* < 0.05) had shorter latencies to the hidden platform than CCI + VEH mice (Supplemental Fig. 1A). Sham + VEH mice (D15-D18 and VP; *p* < 0.05) and CCI + sgp130-Fc mice (D14-17; *p* < 0.05) spent less time in the peripheral zone versus CCI + VEH mice (Supplemental Fig. 1B). CCI + VEH mice had increased path lengths versus Sham + VEH and CCI + sgp130-Fc mice (D15; *p* < 0.05; Supplemental Fig. 1C).

Overall, these data show that CCI impaired learning and memory in the MWM task compared to sham procedures. Additionally, mice treated with sgp130-Fc after CCI had improved MWM performance (decreased latency and path length) compared to injured control (VEH-treated) mice during the testing days most proximate (D14-15) to the last dose of sgp130-Fc. sgp130-Fc treatment after CCI also reduced anxiety-like behavior (decreased peripheral zone time) compared to injured controls.

#### sgp130-Fc dose response on MWM outcomes

**Fig. 3:**
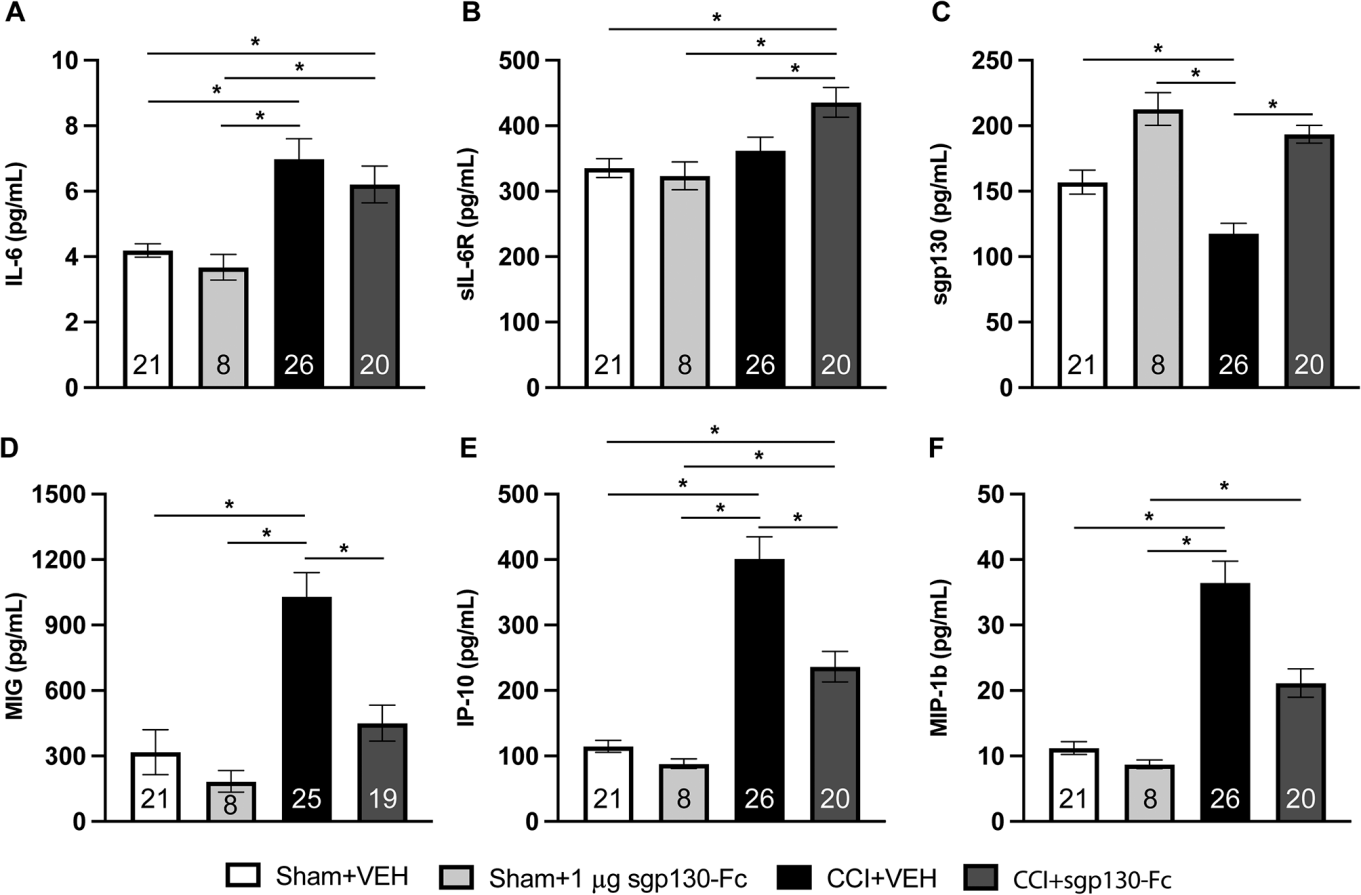
Brain levels of IL-6 related biomarkers and inflammatory chemokines 21 days post CCI. (**a**) IL-6, (**b**) sIL-6R, (**c**) sgp130, (**d**) MIG, (**e**) IP-10, (**f**) MIP-1β were analyzed with Kruskal-Wallis tests and Dunn’s Test. Significant comparisons (*) indicate *p* < 0.05. Bars represent mean ± SEM

Our initial study design compared the effect of two doses of sgp130-Fc after CCI (0.25 µg and 1 µg) on outcome. Prior to combining the 0.25 µg and 1 µg sgp130-Fc groups for Figs. 2 and 3 and Supplemental Fig. 1 analysis, we assessed our data for group differences in the behavioral and biomarker assays based on treatment dose. No statistical differences were noted for speed adjusted or non-adjusted MWM outcomes (latency, peripheral zone time, path length, mean speed) between the two treatment groups (Supplemental Figs. 2, 3 and 4). There was a non-significant effect observed for platform latencies (Supplemental Fig. 2B, 3 A) and peripheral zone times (Supplemental Fig. 2C, 3B) in that mice treated with the higher sgp130-Fc dose (1 µg) had numerically better outcomes than mice treated with the lower sgp130-Fc dose (0.25 µg).

### Neuroinflammation

#### IL-6 related biomarkers

Brain IL-6, sIL-6R, and sgp130 levels were measured (D21) in the hemisphere ipsilateral to the injury site. CCI + VEH mice (*p* < 0.001) and CCI + sgp130-Fc mice (*p* < 0.05) had increased IL-6 levels versus Sham + VEH mice. sgp130-Fc treatment had no impact on brain IL-6 levels (Fig. 3A). sIL-6R levels were similar between CCI + VEH, Sham + VEH and Sham + sgp130-Fc groups; however, CCI + sgp130-Fc mice had higher sIL-6R levels (*p* < 0.05) than Sham + VEH mice (Fig. 3B). sgp130 levels were decreased in CCI + VEH mice versus Sham + VEH and Sham + sgp130-Fc mice. sgp130 was increased in CCI and Sham mice treated with sgp130-Fc (CCI + sgp130-Fc and Sham + sgp130-Fc) versus CCI + VEH mice (*p* < 0.0001) (Fig. 3C).

#### IL-6 related chemokines

The IL-6 associated chemokines, monokine induced by gamma (MIG, CXCL9), interferon γ-induced protein 10 kDa (IP-10, CXCL10) and macrophage inflammatory protein-1β (MIP-1β, CCL4) [17, 38] were also measured (D21) in the hemisphere ipsilateral to the contusion [17, 38]. Chemokine levels were increased in CCI + VEH versus shams (*p* < 0.0001; Fig. 3D-F). sgp130-Fc treatment after CCI (CCI + sgp130-Fc) reduced MIG (*p* < 0.05; Fig. 3D), IP-10 (*p* < 0.05; Fig. 3E), with a trend toward a reduction in MIP-1β (*p* = 0.1001; Fig. 3F) compared to CCI + VEH-treated mice. There were no sgp130-Fc dose effects (0.25 µg vs. 1 µg) after CCI on IL-6 related biomarkers (Supplemental Fig. 4). Interestingly, though, the higher dose of sgp130-Fc (1 µg) resulted in non-significantly elevated IP-10 and MIP-1β levels compared to the lower dose of sgp130-Fc (0.25 µg; Supplemental Fig. 4E-F).

Overall, these data show that sgp130-Fc treatment increased brain levels of sIL-6R (in CCI + sgp130-Fc) and increased sgp130 levels (in CCI + sgp130-Fc and Sham + sgp130-Fc) on D21. Also, CCI resulted in a persistent increase in IL-6 associated chemokines, and treatment with sgp130-Fc attenuated this injury response.

#### IL-6 related biomarkers and chemokine correlations

**Fig. 4:**
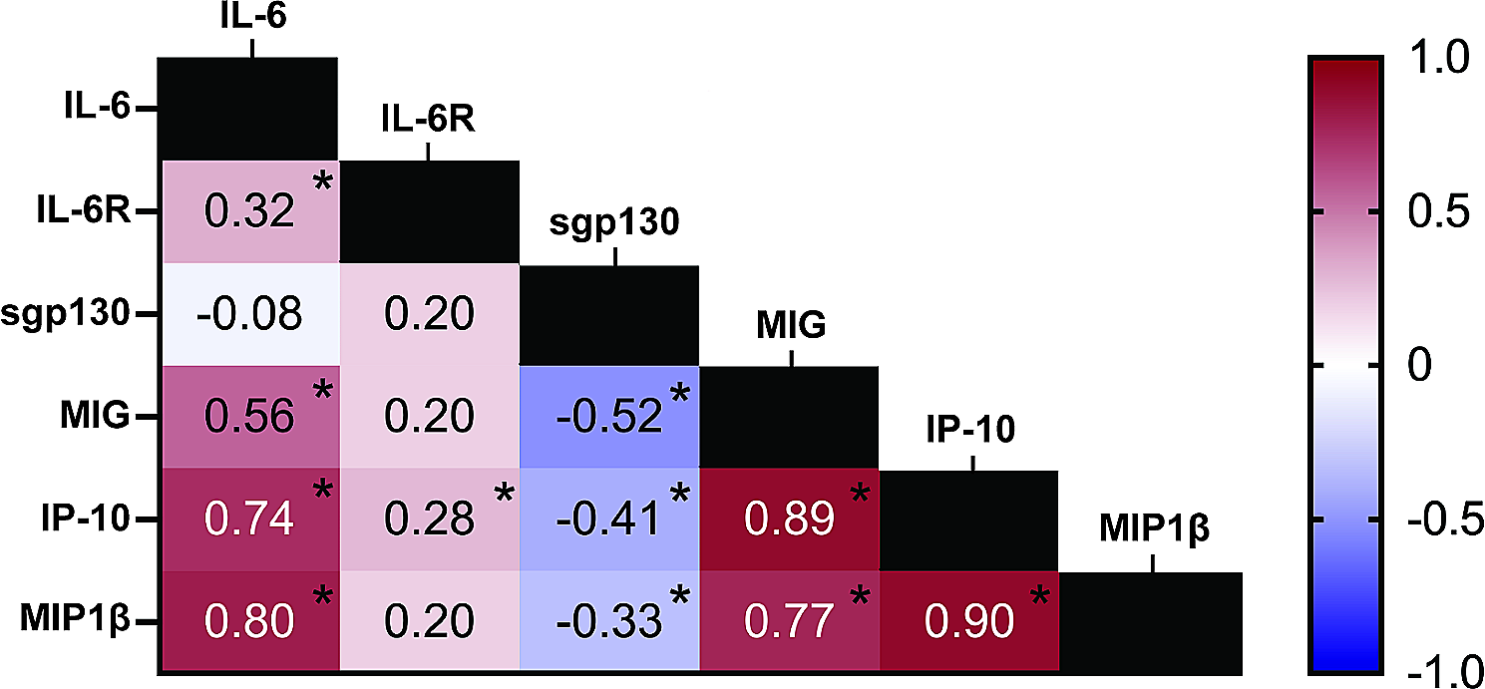
Biomarker correlation heat map. The data shown are Spearman (r) correlations. * indicates statistical significance

We evaluated IL-6 family biomarker relationships with chemokines (Spearman r correlations). IL-6 positively correlated with MIG (*R* = 0.56, *p* < 0.0001), IP-10 (*R* = 0.74, *p* < 0.0001), and MIP-1β (*R* = 0.80, *p* < 0.0001). sIL-6R also positively correlated with MIG (*R* = 0.20, *p* = 0.089), IP-10 (*R* = 0.28, *p* = 0.017), and MIP-1β (*R* = 0.20, *p* = 0.083), but to a lesser extent than IL-6. sgp130 negatively correlated with MIG (*R*=-0.52, *p* < 0.0001), IP-10 (*R*=-0.41, *p* = 0.0003), and MIP-1β (*R*=-0.33, *p* = 0.0037). MIG, IP-10, and MIP-1β were highly correlated with one another (Fig. 4). Together, these findings begin to create a foundation for biomarker-based IL-6 readouts, independent of treatment.

## Discussion

Neuroinflammation impairs recovery post-TBI. IL-6 trans-signaling is a dominant mechanism driving many forms of CNS pathology making its selective inhibition, via sgp130-Fc, a promising therapeutic to promote neurorecovery. Similar to clinical TBI studies, our mouse CCI model showed persistent IL-6 pathology in the setting of neurological deficits [1, 39]. We showed that intermittent systemic treatment with sgp130-Fc after CCI improved learning and decreased anxiety-like behaviors in the MWM. Sgp130-Fc treatment also increased brain sgp130 and sIL-6R and decreased CCI-induced IL-6 family inflammatory chemokine levels when measured 7 days after the last dose given. Overall, our data suggest significant CNS effects after systemic sgp130-Fc administration in reducing IL-6 associated brain tissue damage and functional impairments.

Clinically, IL-6 inhibition promotes anti-inflammatory effects, and inhibitors are used in various disorders including RA, COVID-19 and cytokine release syndrome [40]. The anti-IL-6R antibody, Tocilizumab, is cardioprotective in cardiac arrest patients. In that single-center trial, Tocilizumab reduced systemic inflammation after cardiac arrest, evidenced by decreased C-reactive protein and leukocyte levels [41]. IL-6 signaling inhibition may also be relevant to TBI pathology. Yang et al., demonstrated that pan-IL-6 inhibition improved TBI-induced motor deficits and decreased pro-inflammatory cytokines in a mild closed head injury + hypoxia model [42]. However, there are clinical reports showing that long-term use of Tocilizumab is associated with leukoencephalopathy and also worsened depression [43, 44].

Despite some potential promise, the effect of pan-IL-6 inhibition in the CNS after acute brain injury remains unclear and requires a greater focus on neurologic endpoints [45]. Given the promising results observed with pan-IL-6 inhibition, selective CNS sIL-6R signaling blockage after TBI with sgp130-Fc offers therapeutic potential and the possibility for a reduced side effect profile. Selectively targeting IL-6 trans-signaling via sgp130-Fc could preserve regenerative and neurotrophic effects while reducing immune suppression that is typically associated with suppressed classical IL-6 signaling [23, 46].

We found that two different doses of sgp130-Fc similarly improved CCI-induced functional deficits in that CCI + sgp130-Fc mice had decreased escape latencies and swim distance (MWM) versus CCI + VEH mice. These findings suggest that after CCI, systemic sgp130-Fc administration improves neurorecovery in learning and memory tasks. However, underlying factors like motivation and anxiety appear to affect how sgp130-Fc influences MWM performance. For example, CCI mice had slower swim speeds than Sham + VEH mice. Thus, we adjusted for swim speed variation using a mixed effects regression model which reduced group variability in MWM metrics. CCI + sgp130-Fc mice also had decreased peripheral zone time versus CCI + VEH mice, indicating that IL-6 trans-signaling inhibition may influence injury-induced anxiety-like behaviors. One important relay for anxiety is the hypothalamic pituitary adrenal (HPA) axis. HPA axis stimulation via IL-6 contributes to stress and anxiety following TBI [46]. Our data suggest that the effects of intermittent systemic sgp130-Fc administration on HPA axis-related pathways and associated behaviors after TBI require further study.

Uncontrolled neuroinflammation is also associated with both anxiety and memory impairments [47]. The mediolateral (ML) thalamus, a relay to the amygdala, is involved in MWM-associated thigmotaxis [48], and CCI produces lasting microgliosis in the ML thalamus [49]. Sgp130-Fc treatment-associated reductions in inflammatory chemokines may contribute to improvements in MWM associated anxiety and cognition; however, we evaluated these markers in the hemisphere ipsilateral to the injury and did not perform measurements in distinct brain regions. Thus, investigations into cell-type and region-specific inflammatory patterns associated with CCI and sgp130-Fc treatment, as well as effector function targets like the IL-6 downstream JAK/STAT pathway, are needed to better understand the molecular processes underlying behavioral improvements [50]. Also, future work should include additional assays capturing a variety of cognitive and affective constructs within our model that reflect learning and memory as well as anxiety, anhedonia, sociability, and PTSD-like behaviors in order to further expand our understanding of the potential of sgp130-Fc as a clinically translatable therapeutic agent to TBI survivors.

Other work shows that pan-IL-6 inhibition reduced injury-induced serum IL-6, keratinocyte-derived chemokine, and MIP-1α levels 24 h after hypoxic brain injury in mice [42]. IL-6 neutralization also reduced brain IL-6 levels and serum neuron-specific enolase in that model [42]. Given this work was an exploratory analysis, we chose specific, readily interpretable inflammatory molecular readouts and behavioral readouts as primary endpoints. As such, while we did not directly use histological techniques to measure the extent of neuronal injury after CCI with/without sgp130-Fc treatment, treatment associated reductions in brain IL-6 related chemokine levels are suggestive of reduced neuroinflammation after CCI. However, studies are needed to explore the impact of sgp130-Fc after TBI on CNS specific biomarker burden as well as other region-specific histological outcomes.

Systemic sgp130-Fc administration increased brain sgp130 and sIL-6R, but not IL-6, suggesting that sgp130-Fc may effectively enter the brain possibly due to blood brain barrier disruption and/or other mechanisms like transcytosis, to directly impact CNS damage. Also, systemic IL-6 signaling may modulate local immune cell chemokine production to facilitate T-cell and macrophage infiltration, which may also impact CNS damage [17, 38, 51]. Moreover, IL-6 also stimulates the HPA axis and cortisol production, which may have negative effects on CNS repair and recovery after acute stress and trauma [52] as evidenced by acute cortisol associations with cognitive impairment after severe TBI [53]. Thus, in addition to direct CNS effects, peripheral modulation of IL-6 production via systemic sgp130-Fc administration may impact multiple mechanisms of CNS damage, particularly at later time points in our intermittent dosing regimen when blood brain barrier permeability is not a prominent component of TBI pathology.

Compensatory increases in sIL-6R bioavailability may be reactionary to the intermittent dosing that ended seven days prior to tissue collection, however, increased bioavailability within an appropriately facilitatory local environment may possibly support recovery mechanisms such as neurogenesis, wherein neuronal trans-signaling reportedly has a role [54, 55]. One consideration regarding D21 sIL-6R expression is that the Milliplex assay measures total IL-6R concentrations and cannot differentiate IL-6 bound sIL-6R versus unbound sIL-6R [56]. Thus, further evaluation of the bound vs. unbound sIL-6R is needed. Also, the assay chosen specifically quantified total sIL-6R, which is likely more impacted by sgp130-Fc treatment given its selectivity for trans-signaling [57]; however, future work directly comparing membrane bound IL-6R levels is warranted [12]. Given that sgp130-Fc has a 72-hour half-life in vivo [24], the direct effect on IL-6 associated biomarkers is unknown. However, intermittent systemic sgp130-Fc dosing reversed CCI-induced increases in IL-6 sensitive chemokines MIG, IP-10, and MIP-1β, which are known to perpetuate neuroinflammation in other neurological diseases like encephalitis, MS, and Alzheimer’s disease [17], where IL-6 signaling also plays a role. IL-6 associated inflammation may impact recovery and susceptibility to secondary conditions post-TBI, including cognitive performance deficits, which are reported in our clinical population [39].

The beneficial effects of sgp130-Fc did not appear to be restricted to only after CCI. Interestingly, we found that sham mice treated with sgp130-Fc (Sham + sgp130-Fc) performed better in the MWM (lower latencies, peripheral zone time, and path length) than VEH-treated shams (Sham + VEH). We administered the highest dose tested in this study (1 µg) to sham animals with no adverse effects. This finding may be due to a CNS response to systemic sgp130-Fc treatment, as demonstrated by the increased sgp130 levels in sgp130-Fc treated shams. However, sgp130-Fc treatment in sham mice, did not further reduce IL-6 associated chemokine (MIG, IP-10, MIP-1β). Sham procedures in mice, specifically craniotomy are often associated with some level of brain injury, and thus these findings in sham treated mice suggest that sgp130-Fc merits testing in models of mild and repetitive mild TBI [55].

We did not observe a significant dose response with sgp130-Fc treatment, which was the basis for combining the mice in those two dosing groups. Only a trend toward slightly improved MWM metrics were observed with our high versus lower dose regimen. An examination in future studies of multilevel dosing for other behavioral, histological as well as brain and spleen molecular endpoints (e.g. RNA sequence techology) is required to ensure an optimal dose titration is obtained and its impacts on region and cell specific heterogeneity with a complex injury model such as CCI [58–61]. Further work elucidating brain region specific influences of classical signaling (e.g. microglial) and trans-signaling (e.g. neuronal) on damage burden, plasticity, and repair mechanisms is also warranted. Additional studies are also needed to determine the pharmacokinetics and dynamics of both systemic and CNS sgp130-Fc as well as additional safety testing for long-term treatment and use.

While we identified beneficial effects of trans-signaling blockade via systemic sgp130-FC administration over the initial two weeks after CCI, it is possible that IL-6 trans-signaling may play a neuroprotective role under certain conditions post-injury. For example, Willis et al. showed that in the context of microglial repopulation, IL-6 trans-signaling may support neurogenesis and improve behavioral function in a mouse CCI model [62]. While the results are intriguing, this model required full microglial turnover to obtain this effect along with a high-dose (2ug) intrahippocampal sgp130-Fc injection [62]. However, our work is consistent with other work demonstrating the detrimental effects of sIL-6R trans-signaling in multiple neurodegenerative disease states, and the beneficial impacts of central sgp130 administration on sickness behaviors and associated cognitive dysfunction [7, 15].

## **Conclusion**

We demonstrated beneficial effects of systemic sgp130-FC administration on behavioral performance and neuroinflammation after severe CCI in mice, with no adverse impacts on mortality. The selective nature of sgp130-Fc targets trans-signaling, leaving classical signaling intact. While this exploratory report suggests possible therapeutic benefit, dose optimization in males and females, further characterization of target engagement and additional neurorecovery outcomes require further examination. Overall, our data suggest that sgp130-Fc may be a promising therapy for post-acute TBI recovery with potential for future clinical applications.

### Electronic supplementary material

Below is the link to the electronic supplementary material.

Supplemental Fig. 1. Effect of sgp130-Fc post-CCI on non-speed-adjusted MWM metrics (a) Latencies to hidden platform (b) Peripheral zone time (c) Path length and during learning acquisition (D14-18) and VP (D19). Acquisition data were analyzed via mixed modeling for main effect and Sidak post-hoc testing. VP was analyzed using linear regression. Black arrow on D13 represents final sgp130-Fc or VEH administration. Lines/bars represent mean ± SEM. Significant comparisons (*p* < 0.05) include: *Sham + VEH vs. CCI + VEH, ^%^Sham + VEH vs. CCI + sgp130-Fc, ^Sham + VEH vs. Sham + 1 µg sgp130-Fc, ^#^CCI + VEH vs. CCI + sgp130-Fc, ^+^Sham + 1 µg sgp130-Fc vs. CCI + VEH, ^!^Sham + 1 µg sgp130-Fc vs. CCI + sgp130-Fc.

Supplemental Fig. 2. Effect of two doses of sgp130-Fc treatment after CCI on MWM metrics. (a) Mean speed (b) speed adjusted escape latencies (c) speed adjusted peripheral zone time (d) speed adjusted path length Learning acquisition data were analyzed via mixed modeling for main effect and Sidak post-hoc testing. VP was analyzed using linear regression. Black arrow on D13 represents final sgp130-Fc or VEH administration. Lines and bars represent mean ± SEM. Significant comparisons (*p* < 0.05) are as follows: *Sham + VEH vs. CCI + VEH, ^%^Sham + VEH vs. CCI + 0.25 µg sgp130-Fc, ^$^Sham + VEH vs. CCI + 1 µg sgp130-Fc, ^^^Sham + VEH vs. Sham + 1 µg sgp130-Fc ^#^CCI + VEH vs. CCI + 0.25 µg sgp130-Fc, ^@^CCI + VEH vs. CCI + 1 µg sgp130-Fc, ^&^CCI + 0.25 µg sgp130-Fc vs. CCI + 1 µg sgp130-Fc, ^!^Sham + 1 µg sgp130-Fc vs. CCI + VEH, ^?^Sham + 1 µg sgp130-Fc + CCI + 0.25 µg sgp130-Fc, ^~^Sham + 1 µg sgp130-Fc + CCI + 1 µg sgp130-Fc.

Supplemental Fig. 3. Effect of two doses of sgp130-Fc after CCI on non-speed adjusted MWM metrics. (a) Non-speed adjusted latencies (b) non-speed adjusted peripheral zone time and (c) non-speed adjusted path length. Learning acquisition data were analyzed via mixed modeling for main effect and Sidak post-hoc testings. VP was analyzed using linear regression. Black arrow on D13 represents final sgp130-Fc or VEH administration. Lines and bars represent mean ± SEM. Significant comparisons (*p* < 0.05) are as follows: *Sham + VEH vs. CCI + VEH, ^%^Sham + VEH vs. CCI + 0.25 µg sgp130-Fc, ^$^Sham + VEH vs. CCI + 1 µg sgp130-Fc, ^^^Sham + VEH vs. Sham + 1 µg sgp130-Fc ^#^CCI + VEH vs. CCI + 0.25 µg sgp130-Fc, ^@^CCI + VEH vs. CCI + 1 µg sgp130-Fc, ^&^CCI + 0.25 µg sgp130-Fc vs. CCI + 1 µg sgp130-Fc, ^!^Sham + 1 µg sgp130-Fc vs. CCI + VEH, ^?^Sham + 1 µg sgp130-Fc + CCI + 0.25 µg sgp130-Fc, ^~^Sham + 1 µg sgp130-Fc + CCI + 1 µg sgp130-Fc.

Supplemental Fig. 4. Effect of two doses of sgp130-Fc after CCI on IL-6 related biomarkers and chemokines. (a) IL-6, (b) sIL-6R, (c) sgp130, (d) MIG, (e) IP-10, (f) MIP-1β were analyzed with Kruskal-Wallis tests. Post-hoc comparisons used a Dunn’s Test. Significant comparisons (*) indicated *p* < 0.05. Lines and bars represent mean ± SEM.

## Data Availability

All data generated or analyzed during this study are included in this published article and its supplementary information files.

## Abbreviations

CCI: Controlled Cortical Impact
CNS: Central Nervous System
CSF: Cerebrospinal Fluid
D: Days
Fc: Fusion Protein
FI: Fluorescent Intensity
gp: Glycoprotein
HPA: Hypothalamic Pituitary Adrenal
IBD: Inflammatory Bowel Disease
ICAM: Intercellular Adhesion Molecule
IL: Interleukin
IP: Interferon γ-induced Protein
JAK/STAT: Janus Kinase/Signal Transducer and Activator of Transcription
m: Membrane-bound
MCP: Monocyte Chemoattractant Protein
MHC: Major Histocompatibility Complex
MIG: Monokine Induced by Gamma
min: Minutes
MIP: Macrophage Inflammatory Protein
ML: Mediolateral
MS: Multiple Sclerosis
MWM: Morris Water Maze
LPS: Lipopolysaccharide
PBS: Phosphate-Buffered Saline
R: Receptor
RA: Rheumatoid Arthritis
s: Soluble
sec: Seconds
SEM: Standard Error of the Mean
TBI: Traumatic Brain Injury
TM: Trimmed Mean
VEH: Vehicle
VP: Visible Platform
