# Supplementary figures and images for: Exploratory assessment of the effect of systemic administration of soluble glycoprotein 130 on cognitive performance and chemokine levels in a mouse model of experimental traumatic brain injury
Source: J Neuroinflammation. 2024 Jun 5;21:149. doi: 10.1186/s12974-024-03129-0 (PMC11155101; doi:10.1186/s12974-024-03129-0)

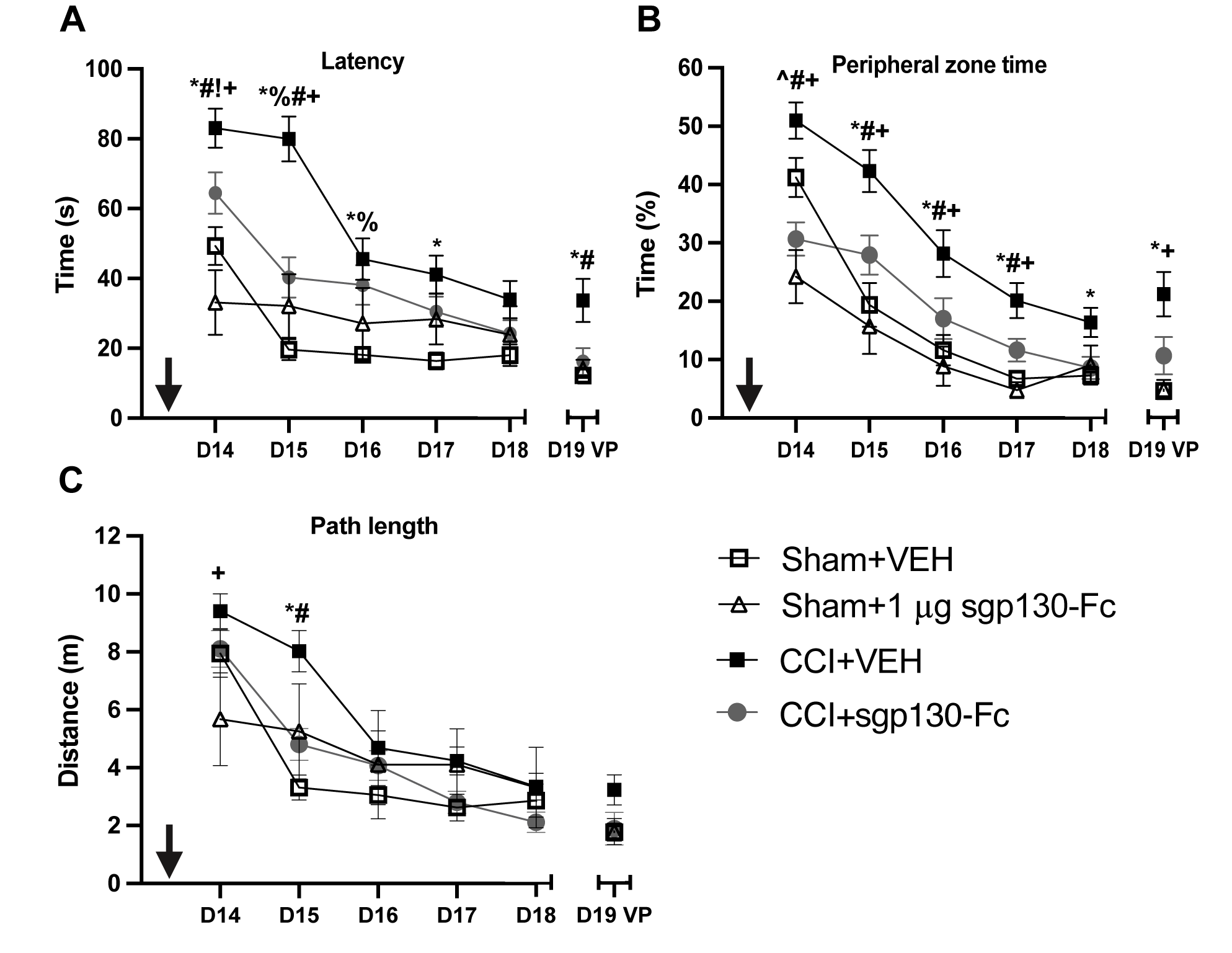

Supplement: Supplementary file 1 — Supplemental Fig. 1. Effect of sgp130-Fc post-CCI on non-speed-adjusted MWM metrics (a) Latencies to hidden platform (b) Peripheral zone time (c) Path length and during learning acquisition (D14-18) and VP (D19). Acquisition data were analyzed via mixed modeling for main effect and Sidak post-hoc testing. VP was analyzed using linear regression. Black arrow on D13 represents final sgp130-Fc or VEH administration. Lines/bars represent mean ± SEM. Significant comparisons (p < 0.05) include: *Sham + VEH vs. CCI + VEH, %Sham + VEH vs. CCI + sgp130-Fc, ^Sham + VEH vs. Sham + 1 µg sgp130-Fc, #CCI + VEH vs. CCI + sgp130-Fc, +Sham + 1 µg sgp130-Fc vs. CCI + VEH, !Sham + 1 µg sgp130-Fc vs. CCI + sgp130-Fc. [file 12974_2024_3129_MOESM1_ESM.png]

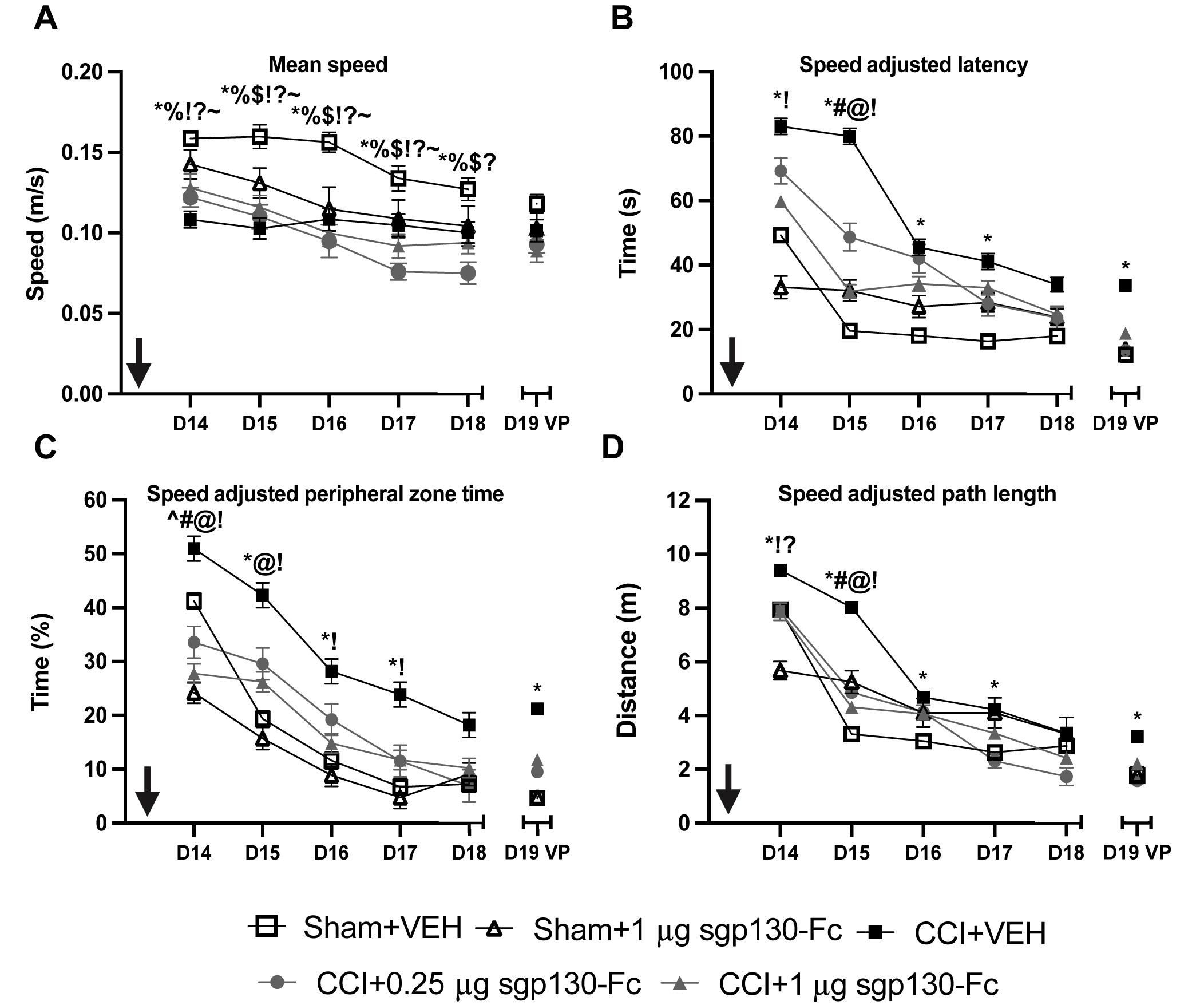

Supplement: Supplementary file 2 — Supplemental Fig. 2. Effect of two doses of sgp130-Fc treatment after CCI on MWM metrics. (a) Mean speed (b) speed adjusted escape latencies (c) speed adjusted peripheral zone time (d) speed adjusted path length Learning acquisition data were analyzed via mixed modeling for main effect and Sidak post-hoc testing. VP was analyzed using linear regression. Black arrow on D13 represents final sgp130-Fc or VEH administration. Lines and bars represent mean ± SEM. Significant comparisons (p < 0.05) are as follows: *Sham + VEH vs. CCI + VEH, %Sham + VEH vs. CCI + 0.25 µg sgp130-Fc, $Sham + VEH vs. CCI + 1 µg sgp130-Fc, ^Sham + VEH vs. Sham + 1 µg sgp130-Fc #CCI + VEH vs. CCI + 0.25 µg sgp130-Fc, @CCI + VEH vs. CCI + 1 µg sgp130-Fc, &CCI + 0.25 µg sgp130-Fc vs. CCI + 1 µg sgp130-Fc, !Sham + 1 µg sgp130-Fc vs. CCI + VEH, ?Sham + 1 µg sgp130-Fc + CCI + 0.25 µg sgp130-Fc, ~Sham + 1 µg sgp130-Fc + CCI + 1 µg sgp130-Fc. [file 12974_2024_3129_MOESM2_ESM.png]

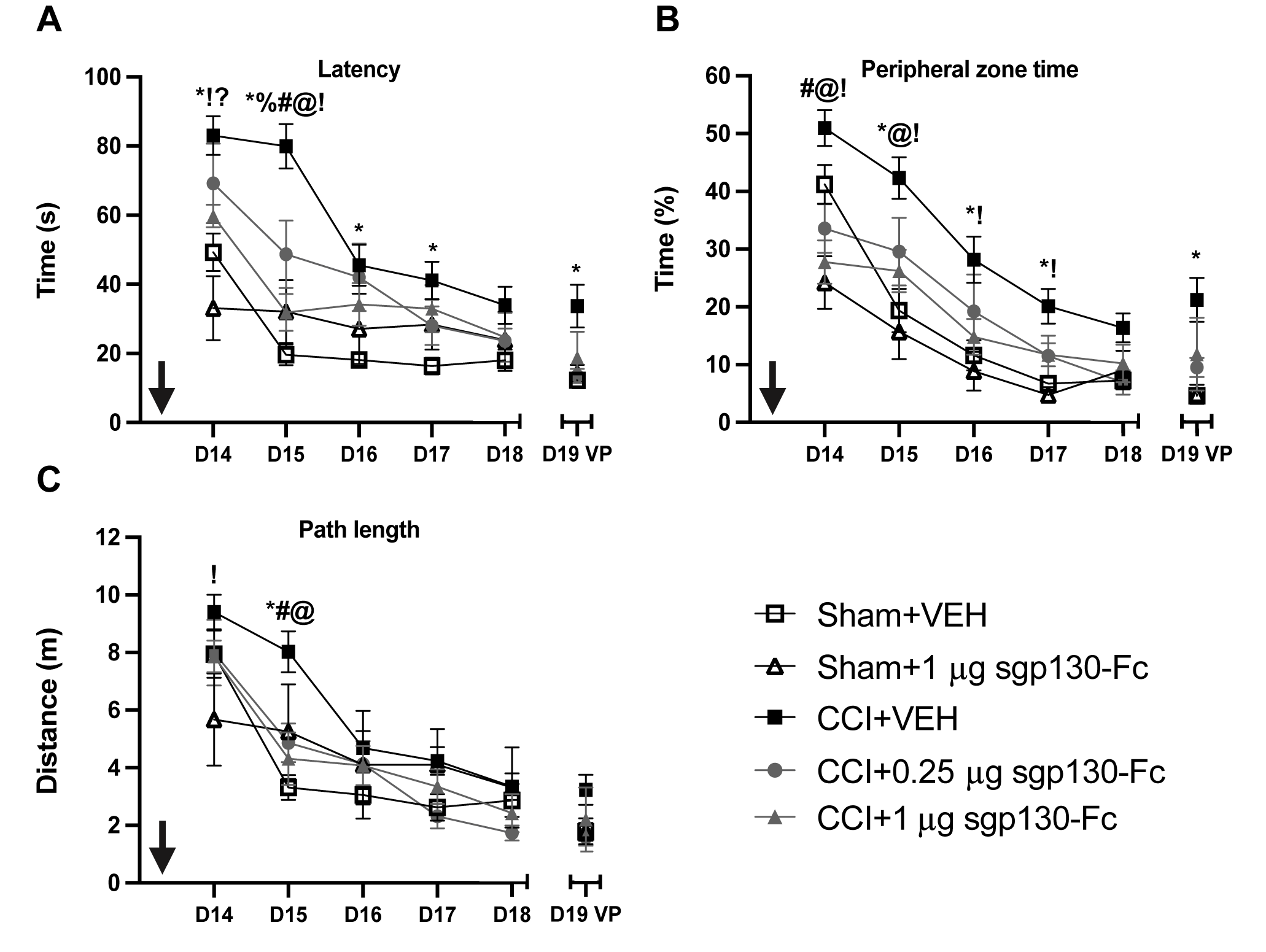

Supplement: Supplementary file 3 — Supplemental Fig. 3. Effect of two doses of sgp130-Fc after CCI on non-speed adjusted MWM metrics. (a) Non-speed adjusted latencies (b) non-speed adjusted peripheral zone time and (c) non-speed adjusted path length. Learning acquisition data were analyzed via mixed modeling for main effect and Sidak post-hoc testings. VP was analyzed using linear regression. Black arrow on D13 represents final sgp130-Fc or VEH administration. Lines and bars represent mean ± SEM. Significant comparisons (p < 0.05) are as follows: *Sham + VEH vs. CCI + VEH, %Sham + VEH vs. CCI + 0.25 µg sgp130-Fc, $Sham + VEH vs. CCI + 1 µg sgp130-Fc, ^Sham + VEH vs. Sham + 1 µg sgp130-Fc #CCI + VEH vs. CCI + 0.25 µg sgp130-Fc, @CCI + VEH vs. CCI + 1 µg sgp130-Fc, &CCI + 0.25 µg sgp130-Fc vs. CCI + 1 µg sgp130-Fc, !Sham + 1 µg sgp130-Fc vs. CCI + VEH, ?Sham + 1 µg sgp130-Fc + CCI + 0.25 µg sgp130-Fc, ~Sham + 1 µg sgp130-Fc + CCI + 1 µg sgp130-Fc. [file 12974_2024_3129_MOESM3_ESM.png]

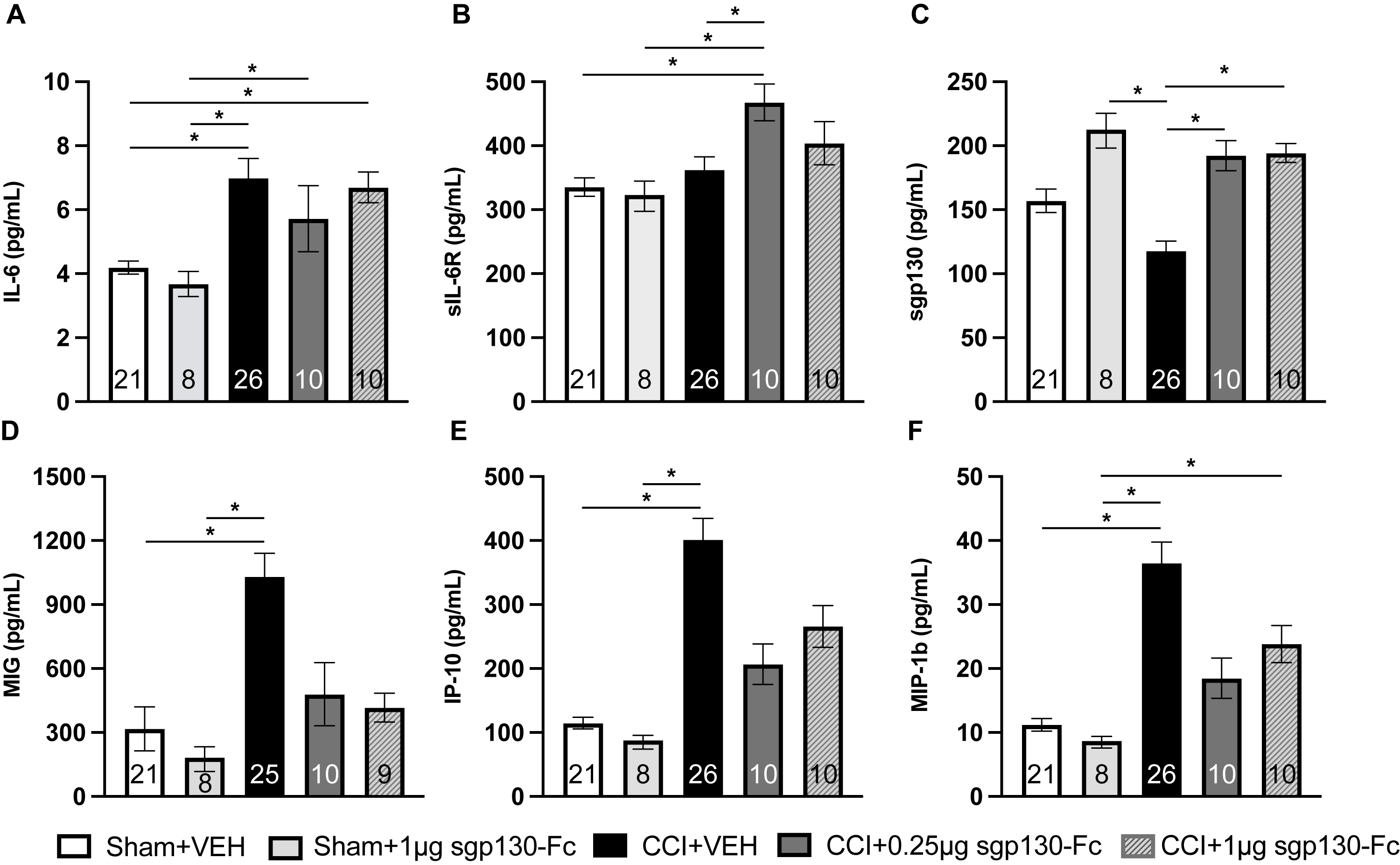

Supplement: Supplementary file 4 — Supplemental Fig. 4. Effect of two doses of sgp130-Fc after CCI on IL-6 related biomarkers and chemokines. (a) IL-6, (b) sIL-6R, (c) sgp130, (d) MIG, (e) IP-10, (f) MIP-1β were analyzed with Kruskal-Wallis tests. Post-hoc comparisons used a Dunn’s Test. Significant comparisons (*) indicated p < 0.05. Lines and bars represent mean ± SEM. [file 12974_2024_3129_MOESM4_ESM.png]
